# Association of gestational metabolic syndrome with the Chinese Healthy Eating Index in mid-pregnancy: a cross-sectional study

**DOI:** 10.1186/s12986-024-00780-5

**Published:** 2024-01-26

**Authors:** Hui Wu, Min-hui Yi, Bing-gang Liu, Yan Xu, Qin Wu, Yu-hong Liu, Ling-peng Lu

**Affiliations:** 1 Department of Nutrition, Seventh People’s Hospital of Shanghai University of Traditional Chinese Medicine, Shanghai, 200137 China; 2 Department of Obstetrics and Gynecology, Seventh People’s Hospital of Shanghai University of Traditional Chinese Medicine, Shanghai, 200137 China; 3 Department of Clinical Lab, Seventh People’s Hospital of Shanghai University of Traditional Chinese Medicine, Shanghai, 200137 China

**Keywords:** Chinese healthy diet index, Cross-sectional survey, Dietary frequency questionnaire, Gestational metabolic syndrome, Mid-pregnancy

## Abstract

**Background:**

This study aims to investigate the relationship between gestational metabolic syndrome (GMS) and the Chinese Healthy Eating Index (CHEI) in mid-pregnancy, and to identify potentially beneficial or high-risk dietary habits. We have developed a mid-pregnancy version of CHEI-2022, adapting the Chinese Healthy Eating Index to align with the food quantity recommendations outlined in the 2022 Dietary Guidelines for Chinese Residents for mid-pregnancy.

**Methods:**

Using the inclusion and exclusion criteria, data from 2411 mid-pregnant individuals were collected through interviews. The Total CHEI score and its component scores were determined through analysis of responses from the food frequency questionnaire. GMS diagnosis involved conducting physical examinations and performing blood biochemical tests. A logistic regression model was employed to analyze the relationship between GMS or related indices and both the total CHEI score and its component scores.

**Results:**

The study identified an overall GMS prevalence of 21.65% (522 out of 2411 participants). During mid-pregnancy, participants diagnosed with GMS exhibited higher BMI, FBG, 1hPBG, 2hPBG, TC, TG, HDL, SBP, as well as higher educational levels and daily activity, compared to those without GMS (*P* < 0.001). After adjusting for potential confounders, participants with higher total CHEI scores (≥ 80) were found to have lower odds of GMS or related indices (*P* < 0.05). Increasing dietary intake of potatoes, whole grains, beans, dark green vegetables, and fruits, as per the CHEI recommendations, was associated with reduced odds of GMS or related indices (*P* < 0.05).

**Conclusion:**

A high-quality diet, as indicated by a total CHEI score of 80 or higher, and increased consumption of specific dietary components, namely potatoes, beans, dark green vegetables, and fruits, were found to effectively reduce the odds of GMS or related indices during mid-pregnancy.

## Background

Gestational metabolic syndrome (GMS) is an independent pathological condition during pregnancy, characterized by various metabolic abnormalities: overweight/obesity, insulin resistance (IR), dysregulated lipid and glucose metabolism, and elevated blood pressure. This syndrome can lead to multiple gestational complications, such as preterm delivery, larger-than-gestational-age infants, smaller-than-gestational-age infants, and fetal growth restriction. Furthermore, GMS offers an opportunity for the timely prevention and management of cardiovascular disease and type 2 diabetes in women [1]. Consequently, there has been an increased focus in recent years on the prevention and screening of metabolic diseases during pregnancy. These findings underscore the importance of rational eating patterns in the prevention and management of GMS. For instance, anti-inflammatory diets have been demonstrated to mitigate gestational complications associated with GMS [2]. Consequently, dietary factors are increasingly recognized as significant predictors in the onset and progression of chronic diseases.

When investigating the relationship between diet and disease, assessing dietary status becomes crucial. In previous studies, there has been a particular focus on examining the association between specific foods, nutrients, and disease [3–5]. Focusing solely on one food can lead to confounding effects from other dietary factors and may not capture the full complexity of the diet. Consequently, studies that focus on specific foods or nutrients often yield controversial results. As a result, studies examining dietary patterns have gained increasing attention. Dietary pattern research employs two primary approaches: data-driven patterns determined through factor and cluster analysis, and scoring-based patterns. The latter approach is grounded in dietary guidelines or other scientific guidance, using recommended food and nutrient intake to establish a straightforward and practical comprehensive index. Two well-established index scores are the Dietary Quality Index (DQI) [6] and the Healthy Eating Index (HEI) [7]. HEI serves as an assessment tool to gauge the extent to which residents adhere strictly to the Dietary Guidelines for Americans in terms of overall dietary quality.It employs a continuous scoring system based on the consumption of foods recommended by the Dietary Guidelines for Americans, simplifying interpretation and facilitating statistical analysis. Over time, as dietary guidelines have been revised, the components of the HEI scoring system have also been adjusted to align with the evolving requirements for evaluating dietary quality, leading to versions like HEI-2005 and HEI-2010. However, it is important to note that various countries, including Canada, Bama, Australia, and others [8], have tailored the components of the HEI scoring system in accordance with their local dietary guidelines, considering the variations in dietary habits and nutritional status. These scoring systems not only assess dietary quality but are also utilized to analyze the association between dietary habits and specific health outcomes.

Following the 2016 revision of the Dietary Guidelines for Chinese Residents (DGC), Yuan et al. developed the Chinese Healthy Eating Index (CHEI), basing it on the HEI [8]. The CHEI has been employed to investigate the relationship between dietary patterns and various conditions, such as breast cancer [9], liver cancer [10], and metabolic syndrome [11]. However, its application in the context of GMS remains unexplored. Considering the recent revision of DGC-2022 in China, which builds upon DGC-2016, and the prevalent clinical diagnosis of GMS during mid-pregnancy, our study focuses on this vital phase. We developed a version of CHEI-2022 for mid-pregnancy, adapted from the original CHEI, and aligned with the dietary recommendations of DGC-2022 for this specific stage of pregnancy. Additionally, our study assessed the dietary qualities during mid-pregnancy and their association with GMS.

## Methods

### Participants

We recruited a total of 2527 women in mid-pregnancy (24–28 weeks) who were undergoing regular obstetric checkups at the Seventh People's Hospital of Shanghai University of Traditional Chinese Medicine between January 2021 and May 2022. The inclusion criteria were singleton pregnancy, adequate language expression and comprehension, and informed consent for study participation. Exclusion criteria included a prior diagnosis of diabetes mellitus, polycystic ovary syndrome, hypertension, thyroid disease, acute and chronic infectious diseases with evident signs of infection at the time of the study, or other major diseases. Informed consent was obtained from all participants in the study.

### Data collection and biochemical parameter detection

General information gathered during mid-pregnancy comprised age (calculated as the year of the survey minus the year of birth), educational level, employment status, etc. According to DGC-2022, more than 30 min of moderate-intensity exercise daily is recommended as the ideal exercise intensity during mid-pregnancy. Pre-pregnancy BMI was ascertained using the BMI recorded during the obstetric examination before 12 weeks of gestation. Blood pressure measurements, comprising systolic (SBP) and diastolic (DBP), followed the methods outlined in WS/T 424–2013 "Anthropometric Methods for Population Health Surveillance." Each measurement was taken three times, with the mean value used for analysis. In China, especially in first-tier cities such as Shanghai, a significant emphasis is placed on promoting nutrition and healthy lifestyles during pregnancy. Pregnant women receive regular information on healthy pregnancy practices during prenatal checkups and early pregnancy. Consequently, in the surveyed population, only three pregnant women reported smoking and drinking alcohol during pregnancy, constituting less than 0.05% of the total sample, and were therefore considered negligible.

During regular obstetric checkups in mid-pregnancy, 2 ml of fasting venous blood was routinely collected, thus eliminating the need for additional venous blood draws from the participants. Serum was subsequently separated using a standardized operating procedure and stored at -80℃ for future testing and analysis. All samples were collected collectively and batch-tested simultaneously. The parameters of fasting blood glucose (FBG), 1-h postprandial blood glucose (1hPBG), 2-h postprandial blood glucose (2hPBG), total cholesterol (TC), triglycerides (TG), high-density lipoprotein (HDL), and low-density lipoprotein (LDL) were measured using a fully automated biochemical analyzer (AU5800, Beckman Coulter, Brea, CA, USA). The measurements of FBG, 1hPBG, and 2hPBG employed the glucokinase method, while TC, TG, HDL, and LDL were assessed using the enzymatic method. Stringent quality control measures were applied to all tests prior to sample analysis.

### Diagnostic criteria for GMS

GMS diagnosis accounted for the characteristics of the Chinese physique, physiological insulin resistance (IR), and metabolic changes during pregnancy. The diagnosis employed the diagnostic criteria for metabolic syndrome established by the Chinese Medical Association Diabetes Society in 2004 [12], augmented by additional reference to Wiznizer et al. [13]. Diagnostic criteria for GMS in this study were as follows: (1) Pre-pregnancy BMI ≥ 24 kg/m2, suggesting overweight or obesity. (2) Gestational diabetes mellitus (GDM): Per China's 2014 guidelines for GDM diagnosis and treatment [14], a 75 g oral glucose tolerance test (OGTT) is administered, with a diagnosis confirmed upon reaching or exceeding any of the following values: FBG ≥ 5.1 mmol/L, 1hPBG ≥ 10.0 mmol/L, or 2hPBG ≥ 8.5 mmol/L. (3) Hypertension: Defined as blood pressure ≥ 140/90 mmHg. (4) Dyslipidemia: Characterized by TG levels ≥ 3.23 mmol/L [1]. It is noteworthy that TG is an independent, significant risk factor for GMS, with a diagnostic threshold of ≥ 3.23 mmol/L (75th percentile among ≥ 1245 healthy pregnant women), applicable in instances of both preeclampsia and gestational diabetes [1]. GMS diagnosis occurred when three or all of the aforementioned criteria were met.

### Dietary questionnaire

The dietary questionnaire employed the dietary review method, involving face-to-face interviews and the use of food models to gather dietary intake information from pregnant women since the beginning of their pregnancy. The Food Frequency Questionnaire (FFQ) was supplied by the research group, and its relative reliability and validity were assessed during the preliminary investigation to ensure the research's overall reliability. Based on the classification principles in the Chinese Food Composition List (sixth edition) [15], foods were categorized into 19 groups. The brand, frequency, and quantity of multivitamin intake were recorded. These data were then processed to estimate daily multivitamin consumption. In the FFQ, a scale ranging from 0 to 10 gauged taste preferences (such as from very salty to very light, and from very oily to very light), indicative of individual salt and oil intake. Only 10 pregnant women reported the consumption of coffee and tea, representing less than 0.05% of the total survey population and were therefore deemed negligible.

Subjects were excluded for incomplete dietary questionnaires, missing information on oils and condiments, or if they reported energy intakes below 500 kcal/day or above 3500 kcal/day, attributable to potential errors in questionnaire responses or survey bias. Of the 2411 subjects ultimately included in the analysis, 522 were diagnosed with GMS, and 1889 were not.

### Calculation of CHEI

The CHEI converts the intake and number of servings of each food group as recorded in the FFQ into scores for 17 components, each bearing a maximum score of either 5 or 10 points, cumulatively adding up to 100 points. The CHEI-2022 version, employed in this study, represents an adaptation of the CHEI-2016 and aligns with the DGG-2022 food recommendations for mid-pregnancy. It was divided into three major categories: (1) "adequate" intake, comprising coarse cereals, potatoes, fruits, and vegetables; (2) "moderate" intake, comprising fish, shrimps, livestock and poultry meat, eggs; (3) "limited" intake, comprising oils, salt, and sugar. Initially, all 17 components were considered equally, with each being assigned a maximum of 5 points, under the assumption of equal importance. This approach aligns with the DGC-2022 directive which emphasizes the necessity and non-replaceability of all recommended food groups. Oils, sodium, and fruits received additional weighting due to the excessive intake of oils and sodium, typical of the long-term Chinese diet, and associated with various adverse health outcomes, in addition to their widespread consumption at most meals. Given the more profound and lasting health effects of chronic overconsumption of cooking oils and sodium, relative to other food groups, both were assigned a score of 10 points each. Fruits, considered equally vital as vegetables for health, also received 10 points, reflecting the maximum score for vegetables. Consequently, the cumulative score for all 17 components amounted to 100 points.

The CHEI-2022 was employed to evaluate dietary quality during mid-pregnancy, using an equal-weighted continuum scoring system where higher scores indicate superior dietary quality.

The specific criteria and corresponding scores are delineated in Table 1. To provide timely feedback on dietary quality and enhance diet education for individuals in mid-pregnancy, conveying information in an easily understandable manner is imperative, thereby minimizing comprehension bias. Consequently, in lieu of a statistical trichotomy approach, we divided the total CHEI score into three culturally contextual grades, corresponding with traditional Chinese customs: fail (0–60 points), pass (60–80 points), and excellent (80–100 points). This grading system facilitates a simplified comprehension of dietary quality levels for individuals in mid-pregnancy.

**Table 1:** Components and evaluation methods of China Healthy Diet Index

CHEI components	DGG-2022 recommended intakes	Criteria for minimum values (0)	Criterion of maximum value	Maximum value
The percentage of total energy supplied by carbohydrates^†^	50–65%	0% or 100%	50–65%	5
Whole grains as a percentage of carbohydrate > 1/3^†^	75–100 g/d	0 g/d	≥ 87.5 g/d	5
Potatoes^†^	75 g/d	0 g/d	≥ 75 g/d	5
Vegetables^†^	400–500 g/d	0 g/d	≥ 450 g/d	5
Dark green vegetables > 2/3^†^	> 267 g	0 g/d	≥ 333 g	5
Seaweeds^†^	100 g/w	0 g/d	≥ 100 g/w	5
Fruits^†^	200–300 g/d	0 g/d	≥ 250 g/d	10
Dairy^†^	300 g	0 g/d	≥ 300 g/d	5
Beans^†^	20 g	0 g/d	≥ 20 g/d	5
Nuts^†^	10 g	0 g/d	≥ 10 g/d	5
Fishes and shrimps^*^	50–75 g/d	0 g/d	≥ 62.5 g/d, < 75 g/d	5
livestock and poultry meats*	50–75 g	0 g/d	≥ 62.5 g/d, < 75 g/d	5
Liver/blood products^*^	20–50 g/w	0 g/d	≥ 35 g/w, < 50 g/d	5
Eggs^*^	50 g	0 g/d	50 g/d	5
Oils^#^	25 g/d	≥ 50 g/d	< 25 g/d	10
Salt^#^	5 g/d	≥ 10 g/d	< 5 g/d	10
Sugar^#^	25–50 g	≥ 50 g/d	< 25 g/d	5

Calculation methods: ^†^"adequate" intake category. *"moderate" intake category. ^#^"limited" intake category

^†,^^*^Using a two-way scoring method, lower than the minimum limit to reduce the score, the score = 5/recommended intake × actual intake; higher than the maximum limit to reduce the same score, the score = 5–5/recommended intake × (actual intake − recommended intake)

^#^Higher than the maximum value and less than twice the maximum value is proportionally reduced points, the score = 10–10/recommended value × (actual intake − recommended value)

### Statistical analysis

Quantitative data adhering to a normal distribution were described as mean ± standard deviation (SD), while categorical data were expressed as absolute numbers and percentages. The Student's t-test was employed to compare quantitative variables, and the Chi-square test (also known as*** χ***^2^ test) was utilized to compare categorical variables. Univariable binary logistic regression was conducted to analyze the relationship between the total CHEI score and GMS or relative indexes, with the total CHEI score as the independent variable and the presence or absence of GMS or relative indexes as the dependent variable. Multivariable binary logistic regression was utilized to assess the association between the total CHEI score or its component scores and GMS, overweight/obesity, GDM, hypertension, and dyslipidemia. Potential confounders incorporated into the adjusted models included age, maternity history, education level, household income, daily activity, and daily multivitamin intake. A *P* value of < 0.05 was deemed statistically significant. All statistical analyses were conducted using SPSS version 23.0 (SPSS, Armonk, NY, USA), and forest diagrams were generated using GraphPad Prism version 8 (Graphpad, SD, CA, USA).

## Results

### General situation

The study revealed an overall GMS prevalence of 21.65% (522 out of 2411). Individuals in mid-pregnancy with GMS demonstrated higher BMI, FBG, 1hPBG, 2hPBG, TC, TG, HDL, SBP, education levels, and daily activity in comparison to those without GMS (*P* < 0.001). Furthermore, energy intake, daily multivitamin use, and DBP exhibited statistically significant differences between groups with and without GMS (*P* < 0.05). Age, weight gain during pregnancy, LDL, maternity history, and household income showed no significant difference between the two groups (*P* > 0.05). The results are delineated in Table 2.

**Table 2:** General demographic characteristics

Demographic characteristics	Not diagnosed with GMS (n = 1,889)	GMS(n = 522)	*P* value
Age (years)^†^	29.170 ± 4.483	29.160 ± 5.096	0.976
Pre-pregnancy BMI (kg/m^2^)^†^	22.359 ± 4.603	26.414 ± 4.811	< 0.001
Weight gain during pregnancy (kg)	8.852 ± 4.021	8.872 ± 4.845	0.985
Energy intake (kcal/d)^†^	1464.086 ± 411.718	1642.902 ± 636.198	0.003
Daily multivitamin intake^*^	801 (66.14)	223 (57.47)	0.002
FBG (mmol/L)^†^	4.407 ± 0.559	4.576 ± 0.636	< 0.001
PBG-1 h (mmol/L)^†^	7.780 ± 1.715	10.045 ± 1.205	< 0.001
PBG-2 h (mmol/L)^†^	6.397 ± 1.427	7.921 ± 1.267	< 0.001
TC (mmol/L)^†^	5.254 ± 1.236	5.563 ± 1.032	< 0.001
TG (mmol/L)^†^	2.215 ± 1.096	3.591 ± 1.104	< 0.001
LDL (mmol/L)^†^	2.800 ± 0.802	2.848 ± 0.798	0.340
HDL (mmol/L)^†^	1.897 ± 0.468	2.155 ± 0.662	< 0.001
SBP (mmHg)^†^	120.920 ± 11.647	124.880 ± 14.481	< 0.001
DBP (mmHg)^†^	71.910 ± 9.562	73.890 ± 10.888	0.001
Maternity history*			0.515
< 1 live birth	616 (50.87)	190 (48.97)	
≥ 1 live birth	595 (49.13)	198 (51.03)	
Degree of education*			< 0.001
Senior high school and below	610 (50.37)	235 (60.57)	
College degree or above	601 (49.63)	153 (39.43)	
Household income*			0.296
< 10,000 CNY	448 (36.99)	155 (39.95)	
≥ 10,000 CNY	763 (63.01)	233 (60.05)	
Daily activity*			< 0.001
≥ 30 min	754 (62.26)	204 (52.58)	
< 30 min	457 (37.74)	184 (47.42)	

^†^Mean ± standard deviation;

*Outside the brackets is the number of cases, inside the brackets is the composition ratio (%)

### Relationship of total CHEI score with GMS or relative indexes

The total CHEI score and its component scores were calculated based on the FFQ and classified into three grades—fail, pass, and excellent—in accordance with Chinese traditional culture. The relationship between the total CHEI score and GMS, along with metabolic indices, was evaluated using univariable binary logistic regression analysis, and the results are delineated in Table 3. Prior to adjusting for potential confounders, all metabolic indices demonstrated a negative correlation with the total CHEI score, except for decreased HDL, elevated LDL, and DBP. Following adjustments in Model 1 for age, maternity history, education level, household income, daily activity, and multivitamin intake, a 10-point increase in the total CHEI score was associated with a 9.0% reduction (95% CI 3.0–15.0%, *P* < 0.001) in the odds of GMS.

**Table 3:** The relationship between the total CHEI score and GMS or relative indexes

	Continuity CHEI^†^		Classification CHEI		
	*OR* (*95%CI*)	*P* value	Pass[60,80) VS Fail [0,60)^*^ OR (95% CI)	Excellent[80,100) VS Fail[0,60) ^#^ OR (95% CI)	*P*_*trend*_ Value
GMS	0.882 (0.879–0.885)	< 0.001	0.734 (0.729–0.739)	0.529 (0.401–0.657)	< 0.001
Model I	0.991 (0.985–0.997)	< 0.001	0.829 (0.717–0.941)	0.765 (0.680–0.850)	
FBG	0.981 (0.974–0.988)	0.030	0.927 (0.806–1.048)	0.887 (0.768–1.006)	0.060
Model I	0.984 (0.978–0.990)	0.072	0.956 (0.835–1.077)	0.912 (0.793–1.031)	
PBG-1 h	0.884 (0.878–0.890)	< 0.001	0.759 (0.639–0.879)	0.663 (0.643–0.683)	< 0.001
Model I	0.886 (0.874–0.898)	< 0.001	0.825 (0.708–0.942)	0.714 (0.598–0.830)	
PBG-2 h	0.958 (0.943–0.973)	< 0.001	0.779 (0.659–0.899)	0.702 (0.583–0.821)	< 0.001
Model I	0.959 (0.943–0.975)	< 0.001	0.841 (0.723–0.959)	0.641 (0.521–0.761)	
TC	0.978 (0.967–0.989)	< 0.001	0.955 (0.832–1.078)	0.846 (0.727–0.965)	0.011
Model I	0.979 (0.968–0.990)	< 0.001	0.983 (0.862–1.104)	0.912 (0.709–1.122)	
TG	0.891 (0.878–0.904)	< 0.001	0.833 (0.715–0.951)	0.712 (0.588–0.836)	< 0.001
Model I	0.894 (0.880–0.907)	< 0.001	0.774 (0.655–0.893)	0.849 (0.732–0.966)	
HDL	0.995 (0.920–1.070)	0.211	1.071 (0.951–1.191)	0.892 (0.767–1.017)	0.308
Model I	0.995 (0.926–1.064)	0.345	1.052 (0.931–1.173)	0.962 (0.842–1.082)	
LDL	0.990 (0.979–1.001)	0.077	1.062 (0.942–1.182)	0.953 (0.835–1.071)	0.389
Model I	0.988 (0.975–1.001)	0.036	1.077 (0.954–1.200)	0.991 (0.874–1.108)	
SBP	0.944 (0.927–0.962)	< 0.001	0.678 (0.561–0.795)	0.592 (0.471–0.713)	< 0.001
Model I	0.946 (0.928–0.964)	< 0.001	0.732 (0.616–0.848)	0.607 (0.499–0.715)	
DBP	0.984 (0.962–1.007)	0.166	1.018 (0.794–1.242)	1.009 (0.827–1.191)	0.643
Model I	0.991 (0.968–1.014)	0.439	0.938 (0.725–1.151)	0.927 (0.670–1.184)	
Overweight/obesity	0.886 (0.882–0.890)	< 0.001	0.706 (0.595–0.817)	0.518 (0.392–0.644)	< 0.001
Model I	0.977 (0.971–0.983)	< 0.001	0.863 (0.764–0.962)	0.693 (0.656–0.730)	
GDM	0.933 (0.924–0.942)	< 0.001	0.679 (0.559–0.799)	0.482 (0.358–0.606)	< 0.001
Model I	0.973 (0.965–0.981)	< 0.001	0.818 (0.693–0.943)	0.619 (0.496–0.742)	
Hypertension	0.898 (0.893–0.903)	< 0.001	0.762 (0.644–0.880)	0.580 (0.454–0.706)	< 0.001
Model I	0.968 (0.962–0.974)	< 0.001	0.894 (0.878–0.910)	0.696 (0.579–0.813)	
Dyslipidemia	0.899 (0.892–0.906)	< 0.001	0.835 (0.723–0.947)	0.609 (0.486–0.732)	< 0.001
Model I	0.975 (0.968–0.982)	< 0.001	0.862 (0.805–0.919)	0.712 (0.643–0.781)	

Model I adjusted for the potential confounders: including age, maternity history, degree of education, household income, daily activities and daily multivitamin intake

^†^The relationship between continuity CHEI score and GMS or relative indexes

^*^Compared with classification CHEI score’s grand is fail, the relationship between classification CHEI score’s grand is pass (60 -80) and GMS or relative indexes

^#^Compared with classification CHEI score’s grand is fail(< 60), the relationship between classification CHEI score’s grand is excellent (≥ 80) and GMS or relative indexes

In terms of CHEI classification, an excellent grade demonstrated a protective effect in comparison to pass and fail grades for all GMS and its relative indexes. Similarly, a pass grade showed protective effects when compared with a fail grade for GMS and its relative indexes, with the exception of FBG, HDL, LDL, and DBP (*P*_*trend*_ < 0.05). Even after adjustments in Model 1, statistically significant protective factors remained for an excellent grade in comparison to a fail grade. These factors included GMS (OR 0.765, 95% CI 0.680–0.850, *P* < 0.001), PBG-1 h (OR 0.714, 95% CI 0.598–0.830, *P* < 0.001), PBG-2 h (OR 0.641, 95% CI 0.521–0.761, *P* < 0.001), TG (OR 0.849, 95% CI 0.732–0.966, *P* < 0.001), SBP (OR 0.607, 95% CI 0.499–0.715, *P* < 0.001), overweight/obesity (OR 0.693, 95% CI 0.656–0.730, *P* < 0.001), GDM (OR 0.619, 95% CI 0.496–0.742, *P* < 0.001), hypertension (OR 0.696, 95% CI 0.579–0.813, *P* < 0.001), and dyslipidemia (OR 0.712, 95% CI 0.643–0.781, *P* < 0.001).

### Relationship of the total CHEI score or its component scores with GMS or relative indexes

**Fig. 1:**
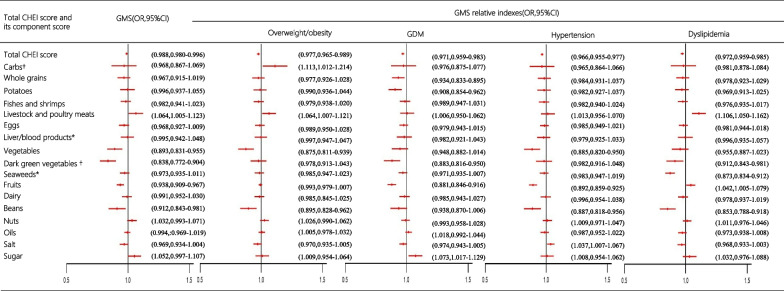
The association between the total CHEI score with GMS by multivariable binary logistic regression. ^†^Indicates the proportion, including the energy supply ratio of carbohydrates and the proportion of dark green vegetables in vegetables; *Indicates unusual break including weekly intake of animal liver/blood product, weekly intake of seaweeds vegetables

The relationship between the total CHEI score, its component score, and GMS or relative indexes is illustrated in Fig. 1. Following adjustments for potential confounding factors, including age, maternity history, degree of education, household income, daily activity, and daily multivitamin intake, the total CHEI score demonstrated negative associations with GMS (OR 0.988, 95% CI 0.980–0.996, *P* = 0.045), overweight/obesity (OR 0.977, 95% CI 0.965–0.989, *P* = 0.039), GDM(OR 0.971, 95% CI 0.959–0.983, *P* = 0.035), hypertension(OR 0.966, 95% CI 0.955–0.977, *P* = 0.027), and dyslipidemia (OR 0.972, 95% CI 0.959–0.985, *P* = 0.036).

Higher CHEI component scores for beans (OR 0.912, 95% CI 0.843–0.981, *P* = 0.011), fruits (OR 0.938, 95% CI 0.909–0.967, *P* = 0.021), vegetables (OR 0.893, 95% CI 0.831–0.955, *P* = 0.016), and the percentage of dark green vegetables in vegetables (OR 0.838, 95% CI 0.772–0.904, *P* < 0.001) were associated with lower odds of developing GMS. Beans (OR 0.895, 95% CI 0.828–0.962, *P* = 0.008) and vegetables (OR 0.875, 95% CI 0.811–0.939, *P* = 0.013) were inversely associated with the odds of overweight/obesity, whereas livestock and poultry meats (OR 1.064, 95% CI 1.007–1.121, *P* = 0.014) and the energy supply ratios of carbohydrates (OR 1.113, 95% CI 1.012–1.214, *P* = 0.006) were positively associated with the odds of overweight/obesity. The percentage of dark green vegetables in vegetables (OR 0.883, 95% CI 0.816–0.95, *P* = 0.012), potatoes (OR 0.908, 95% CI 0.854–0.962, *P* = 0.019), and whole grains (OR 0.934, 95% CI 0.883–0.895, *P* = 0.041) was associated with a reduced likelihood of GDM, while component scores for sugar (OR 1.073, 95% CI 1.017–1.129, *P* = 0.023) were linked to an increased likelihood of GDM. Component scores for vegetables (OR 0.885, 95% CI 0.820–0.950, *P* = 0.012), fruits (OR 0.892, 95% CI 0.859–0.925, *P* = 0.007), and beans (OR 0.887, 95% CI 0.818–0.956, *P* = 0.004) were protective factors against hypertension, whereas salt increased the odds of hypertension (OR 1.037, 95% CI 1.007–1.067, *P* = 0.034).Furthermore, with each 1-point increase in the CHEI beans and seaweeds component scores, the odds of dyslipidemia decreased by 14.7%(95% CI 8.2–21.2%, *P* < 0.001) and 12.7% (95% CI 8.8–16.6%, *P* = 0.009), respectively. Conversely, livestock and poultry meats (OR 1.106, 95% CI 1.050–1.162, *P* < 0.001) and fruits(OR 1.026, 95% CI 1.007–1.081, *P* = 0.011) component scores were positively associated with the odds of dyslipidemia.

## Discussion

A total of 522 mid-pregnant women were diagnosed with GMS, resulting in an overall prevalence of 21.65%, consistent with previous research [16, 17]. Extensive research has elucidated the impact of dietary patterns on GMS, with diets such as the hypertension prevention diet [18], Mediterranean diet [19], and vegetarian diet [20] proving effective in reducing the risk of GMS. This phenomenon may be ascribed to the fact that, even during a typical pregnancy, various inherent physiological alterations predispose individuals to metabolic syndrome. These alterations encompass degrees of IR, adipose tissue accumulation, hyperlipidemia, and an escalation in the systemic inflammatory response [21]. As pregnancy progresses, there is an increase in the secretion of IR-related hormones, including progesterone, estrogen, human placental lactogen, cortisol, and prolactin. As IR progressively exceeds the body's compensatory abilities, it culminates in the manifestation of GMS. Insulin resistance is pivotal in the pathogenesis of GMS [22]. It is hypothesized that a typical pregnancy constitutes a transitory phase of metabolic syndrome (MetS). Although there is a mild systemic inflammatory response, its impact remains benign and does not adversely affect the organism [23]. Metabolic syndrome also contributes to fetal growth by supplying nutrients and acts as a marker of the body's ability to regulate glucolipid and lipid metabolism [24]. However, studies have shown that systemic inflammation can detrimentally affect vascular endothelial function. Moreover, the continuance of certain metabolic disorders after pregnancy is associated with an increased risk of metabolic diseases and cardiovascular events [25]. Consequently, proactive prevention and intervention strategies are essential. Such strategies can reduce the occurrence of pregnancy-related complications and have significant implications for preventing long-term chronic conditions like diabetes, hypertension, and metabolic syndrome in mothers and their offspring post-pregnancy.

It is currently acknowledged that IR constitutes a chronic subclinical inflammatory process [26, 27]. Dietary factors are intricately linked to this process and play a pivotal role in the regulation of subclinical inflammation [28]. For instance, a high-fat diet can significantly elevate gram-negative bacteria in the gut, triggering inflammatory responses and leading to low-level inflammation in the body. Conversely, dietary fiber has the capacity to modulate gut flora, thereby mitigating low-level inflammation [29].Given the protracted and inconspicuous nature of low-level inflammation, it can inflict long-term damage and initiate apoptosis of pancreatic *β*-cells through oxidative stress, ultimately culminating in IR. Consequently, chronic intestinal inflammation and immunity have emerged as significant factors in the development of IR [30].Therefore, an increasing number of studies have emphasized the importance of adopting rational dietary patterns for both the prevention and treatment of GMS. Building upon our previous research, we selected 164 pregnant women in their second trimester and categorized them into two groups based on the outcomes of their OGTT. Dietary intake data were collated through the FFQ, followed by computation of the Dietary Inflammation Index (DII). The results revealed an association between DII and the incidence of GDM. The GDM group exhibited elevated consumption of pro-inflammatory nutrients, including total fat, animal fat, and saturated fatty acids, in contrast to the control group. In contrast, the consumption of anti-inflammatory nutrients, including dietary fiber and polyunsaturated fatty acids, was found to be lower in the GDM group than in the control group [31]. These findings align with the dietary recommendations outlined in the DGC-2022 during Pregnancy. Consequently, this study conducts a more in-depth investigation into the relationship between dietary patterns and GMS, building upon our previous research. The objective was to lay a foundation for evaluating diets and providing dietary advice during pregnancy.

The DGC-2022 advocate for the sufficient consumption of whole grains, potatoes, dark green vegetables, fruits, dairy, and nuts during pregnancy. After adjusting for potential confounding factors, analyses indicated that the intake of beans, fruits, and dark green vegetables significantly decreased the likelihood of GMS. Greater consumption of whole grains, potatoes, dark green vegetables, and fruits correlated with a substantial reduction in the odds of GMS-related indicators, including overweight/obesity, GDM, hypertension, and dyslipidemia. Consistent with findings from previous research, the consumption of dark green vegetables, fruits, and beans exhibited protective effects against GMS [32, 33]. Furthermore, due to the heightened inflammatory state in pregnant women with GMS, pro-inflammatory factors such as tumor necrosis factor-α (TNF-α) and leptin persist at elevated levels post-delivery compared to healthy pregnant women, while anti-inflammatory factors, such as adiponectin, tend to be comparatively diminished [34]. Dark green vegetables, fruits, and beans all comprise anti-inflammatory components of the diet, capable of diminishing systemic inflammation and enhancing IR [35]. Additionally, these dietary components augment the body's antioxidant capacity, inhibit lipid peroxidation, mitigate vascular tone, and enhance endothelial function, thus contributing to reduced blood pressure [36]. Notably, vegetables and beans are low glycemic index foods, and increasing their consumption can aid in blood glucose control [37]. Furthermore, due to their high dietary fiber content, increased consumption of these foods can enhance satiety and reduce the intake of other foods, consequently contributing to body weight reduction [19].

The DGC-2022 advocate for the reduction in the consumption of livestock and poultry meats, oils, salt, and added sugar during pregnancy. After adjusting for potential confounding factors, it is evident that the consumption of livestock and poultry meats correlates with an elevated risk of GMS. Additionally, increased consumption of livestock and poultry meats, sugar, and salt was found to be significantly linked to an increased likelihood of GMS-related parameters, including overweight/obesity, GDM, hypertension, and dyslipidemia. Previous studies have likewise demonstrated a positive correlation between increased livestock and poultry meat intake and an increased prevalence of metabolic disorders [38, 39]. A prospective clinical observation of 2755 cases found that dietary patterns rich in meat were significantly linked to the prevalence of GDM [40]. In a prospective study involving 1868 middle-aged and older adults, research indicated that the consumption of poultry and processed meats may increase the risk of metabolic syndrome. Substituting these meats with other protein-rich foods such as beans, fish, and eggs was found to be potentially effective in averting metabolic syndrome [41]. Numerous studies have reported an association between reduced salt intake and a reduced likelihood of metabolic syndrome [42, 43]. This association may be attributed to the fact that high salt intake decreases the body's baseline aldosterone levels and increases the activity of the salt corticosteroid receptor, thereby contributing to metabolic disruptions [44] by interfering with insulin signaling pathways [45]. Livestock and poultry meats, salt, and added sugar are dietary components with pro-inflammatory properties that promote chronic inflammation within the body. They raise the levels of inflammatory markers, including TNF-α, interleukin (IL)-1β, IL-4, IL-6, and IL-10, thereby exacerbating IR. The pro-inflammatory diet also includes refined carbohydrates, saturated fatty acids, and trans-fatty acids. However, this study did not identify an association between the energy supply ratio of carbohydrates and dietary oil consumption with the likelihood of GMS. This discrepancy may stem from the pregnant women's emphasis on nutritional well-being, characterized by an increased proportion of whole grains in their staple diet, as well as the adoption of soybean oil, corn oil, and olive oil for culinary purposes during pregnancy.

This study demonstrates several strengths and innovations. First, this study utilized a CHEI, aligned with the dietary recommendations outlined in DGC-2022 for mid-pregnancy, to investigate the relationship between dietary patterns and the likelihood of GMS during mid-pregnancy. This approach improves the assessment of dietary quality among Chinese individuals during pregnancy, representing an innovative aspect of this research. Second, the total CHEI score was classified based on traditional Chinese cultural criteria, using cutoff values of 60 and 80. This classification aimed to assess the quality of dietary patterns and offer prompt feedback to pregnant women, thus facilitating dietary education. However, this study presents certain limitations. Firstly, this study utilized a cross-sectional design, which led to lower statistical efficiency compared to a cohort study. Secondly, the FFQ could be influenced by geographic and seasonal variations, and some confounding factors were not taken into account. Thirdly, despite this study being part of a national multi-center project, the data presented in this article are derived from a limited number of patients in a single center. Consequently, these findings only reflect the dietary quality of mid-pregnancy within GaoQiao Town, Pudong New Area, Shanghai. To conduct a nationwide analysis of dietary quality among pregnant women, data from all sub-centers across the nation would need comprehensive integration.

## Conclusion

In conclusion, proper dietary patterns play a significant role in both preventing and treating GMS. The HEI is one of the most frequently employed methods for evaluating dietary patterns. In this study, the use of CHEI-2022, which is based on HEI, proves more effective for analyzing the connection between dietary habits and specific health outcomes in the Chinese maternal population. This study has provided significant evidence, demonstrating that the total CHEI scores and component scores indicate the inclusion of potatoes, fruits, dark green vegetables, and beans in the mid-pregnancy diet as conferring a protective effect against GMS and related indices. Conversely, the consumption of livestock and poultry meat was associated with an increased risk of GMS. Pregnant women are encouraged to adhere to the DGC-2022 (pregnancy version) for dietary optimization during pregnancy. Furthermore, they should strive to maintain an excellent level of overall dietary quality (CHEI ≥ 80) to harness the potential of dietary factors in preventing and treating GMS.

## Data Availability

The data presented in this article are available on request from the corresponding author.

## Abbreviations

GMS: Gestational metabolic syndrome
IR: Insulin resistance
DQI: Dietary quality index
HEI: Healthy dietary index
DGC: Dietary guidelines for Chinese residents
CHEI: Chinese healthy eating index
CNY: China yuan
BMI: Body mass index
SBP: Systolic blood pressure
DBP: Diastolic blood pressure
FBG: Fasting blood glucose
1hPBG: 1-H postprandial blood glucose
2hPBG: 2-H postprandial blood glucose
TC: Total cholesterol
TG: Triglycerides
HDL: High-density lipoprotein
LDL: Low-density lipoprotein
GDM: Gestational diabetes mellitus
OGTT: Oral glucose tolerance test
FFQ: Food frequency questionnaire
Mets: Metabolic syndrome
DII: Dietary Inflammation Index
